# Age-period-cohort analysis of suicide mortality by gender among white and black Americans, 1983–2012

**DOI:** 10.1186/s12939-016-0400-2

**Published:** 2016-07-13

**Authors:** Zhenkun Wang, Chuanhua Yu, Jinyao Wang, Junzhe Bao, Xudong Gao, Huiyun Xiang

**Affiliations:** School of Public Health, Wuhan University, Wuhan, 430071 China; Center for Injury Research and Policy & Center for Pediatric Trauma Research, The Research Institute at Nationwide Children’s Hospital, The Ohio State University, Columbus, OH 43210 USA; Global Health Institute, Wuhan University, Wuhan, 430071 China; College of Medicine, The Ohio State University, Columbus, OH 43210 USA

**Keywords:** Suicide, APC analysis, Race, Gender, United States

## Abstract

**Background:**

Previous studies suggested that the racial differences in U.S. suicide rates are decreasing, particularly for African Americans, but the cause behind the temporal variations has yet to be determined. This study aims to investigate the long-term trends in suicide mortality in the U.S. between 1983 and 2012 and to examine age-, period-, and cohort-specific effects by gender and race.

**Method:**

Suicide mortality data were collected from the Web-based Injury Statistics Query and Reporting System (WISQARS) and analyzed with the Joinpoint regression and age-period-cohort (APC) analysis.

**Results:**

We found that although age-standardized rate of suicide in white males, white females, black males, and black females all changed at different degrees, the overall situation almost has not changed since these changes offset each other. By APC analysis, while the age effect on suicide demonstrate an obvious difference between white males and females (with the peak at 75 to 79 for white males and 45 to 54 for white females), young black people are predominantly susceptible to suicide (risk peaks in early 20s for black males and late 20s for black females). Cohort effects all showed a descending trend, except that in white males and females which showed an obvious increase peaked in around cohort 1960. There was a similar period effect trend between different genders in the same race group, but between the races, differences were found in the period before 1990 and after 2000.

**Conclusion:**

We confirmed that the distinction in age-specific suicide rate patterns does exist by gender and by race after controlling for period and cohort effects, which suggested that minorities’ age patterns of suicide may have been masked up by the white people in the whole population. The differences of period effects and cohort effects between white and black Americans were likely to be mainly explained by the difference in race susceptibility to economic depression.

## Background

Suicide, a serious public health problem worldwide [1], is defined as fatal, intentional, self-inflicted injury with the intent to end life [2]. In the United States, suicide is one of the ten leading causes of death for the whole population and those between the ages of 10 and 64 years [3]. As reported by the Centers for Disease Control and Prevention (CDC), 41,149 Americans committed fatal suicide in 2013, and in the same year, more than 490,000 people were treated in U.S. emergency departments for suicide attempts [4]. Suicide rate is increasing while general mortality rates in the U.S. have been declined [3, 5], and has overtaken car crashes as the leading cause of injury mortality since 2009 [6].

Age, period, and cohort analysis (APC analysis) has been widely used for decades to evaluate the character and nature of patterns in the prevalence/mortality of numerous health problems [7, 8], which could reveal an in-depth understanding of factors behind the observed temporal trends. This can be achieved by estimating the effects of these three time-dependent components on rates separately, which allows the researcher to consider each component independently from the other two. It has already been adopted to evaluate suicide mortality in many developed countries such as Japan [9], England and Wales [7], Spain [10], Sweden [11], and the U.S. [12]. But all these studies only separated their research objects by gender.

Researchers reported that race is also a critical demographic risk factor for suicide in addition to gender [4, 13, 14]. As we know, suicide in the U.S. is ordinarily portrayed as a white phenomenon due to that the vast majority of suicides involve white people (the suicide rate of white people is more than twice as high as the whole nonwhite population [14]). Previous studies suggested that the racial differences in U.S. suicide rates are decreasing, particularly in African Americans, but the cause behind the temporal variations has yet to be determined. How the suicide risk changes across the lifespan among white and black Americans also remains unknown.

The purpose of this study is to investigate the long-term trends in U.S. suicide mortality rate between 1983 and 2012 and to examine age-, period-, and cohort-specific effects by gender among white and black Americans using the age-period-cohort modeling. Since there has been a dearth of suicide trend research in the U.S. by gender and race using APC analysis, the only study we found [15] was completed nearly 30 years ago. It is our hope that the results of this study could not only give clues on resource allocation targeting vulnerable groups for suicide control, but could also provide useful information on individual suicide prevention at different life stages. In addition, our findings might also lay the foundation for a better understanding of the relationship between suicide and the whole complex of social, historical, and ecological factors, thus giving etiological implications on suicide in the U.S.

## Methods

Data used in this study were extracted from the Web-based Injury Statistics Query and Reporting System (WISQARS), an interactive online database that provides fatal and nonfatal injury, violent death, and cost of injury data [16], operated by National Center for Injury Prevention and Control at the U.S. CDC. The data source of WISQARS is a national mortality database compiled by National Centre for Health Statistics. It contains information from death certificates filed in state vital-statistics offices and causes of death reported by attending physicians, medical examiners, and coroners. It also includes demographic information about decedents reported by funeral directors, who obtain that information from family members and other informants. [16] All states have adopted laws that require the registration of deaths and the reporting of fatal deaths, and more than 99 % of the U.S. deaths are registered [17].

There were five race groups in WISQARS: White, Black, American Indian/Alaskan Native, Asian/Pacific Islander and Other (combined). Because the total number of suicide deaths by age groups and gender in the last three race groups was too small for our statistical methods [4], we only selected the White and the Black people as the subjects in this study. Our study period was from 1983 to 2012 due to the data accessibility, and it should be noted that during this period, there was a transition from the 9th to 10th revision of the International Classification of Disease (Codes E950-959 in ICD-9, and X60-84 and Y87.0 in ICD-10). Fortunately, previous research suggested that ICD changes had no substantial impact on the analysis of temporal trends of suicide [18]. Since occurrence of suicide in those under 15 years old is very rare, and evaluations on individuals over 80 involve deaths from other competing causes [19, 20], only rates for those between 15 and 79 years old were considered in this study.

To describe the suicide trends in the U.S., the suicide rates by gender and race were age-standardized using the U.S. 2000 standard population recommended by the National Center for Health Statistics [21]. We used Joinpoint Regression Software (version 4.2.0.1, May 2015) (Statistical Research & Applications Branch, National Cancer Institute, Bethesda, MD, USA) which identified changing points of the trend and estimated the percentage of annual change (PAC). Our study assumption was that the suicide death rates changed at a constant percentage every year change linearly on a log scale, for each time segment. The average percent annual change (APAC), a geometric weighted average of PACs to summarize the trend over certain predetermined fixed interval, was also computed as a summary measure of trend over the whole observation period [22].

To conduct APC analysis, the mortality and population data were arranged in five consecutive 5-year periods from 1983–1997 to 2008–2012 and thirteen five-year age brackets from 15–19 years to 75–79 years. The aim of APC analysis, a statistical tool broadly utilized in the fields of demography, sociology and epidemiology, is to assess the impacts of age, period and cohort on demographic or disease rates. The age effects represent differing risks associated with different age brackets; the period effects represent variations in vital rates over time that are associated with all age groups simultaneously; the cohort effects are associated with changes in rates across groups of individuals with the same birth years—that is, for successive age groups in successive time periods [23]. As there is a linear relationship between the age, period and cohort, it is hard to estimate the unique set for each age, period and cohort effect, which is known as the non-identification problem [23]. Nonetheless, many useful quantities can be estimated. We obtained those parameters by the Age-period-cohort Web Tool (Biostatistics Branch, National Cancer Institute, Bethesda, MD, USA). The PACs for each age group, called “local drifts”, can be generated from log-linear regressions. Other useful estimable parameters such as longitudinal age trend (age trend + period trend) and cross-sectional age trend (age trend − period trend) can be obtained too [24]. The web tool can also calculate the relative rate in any given calendar period (or birth cohort) versus a referent period (or birth cohort), adjusted for age and non-linear cohort (or period) effects [24]. The central age group, period, and birth cohort are often defined as the reference, respectively. In case of an even number of categories, the reference value was set as the lower of the two central values [24].

## Results

**Fig. 1:**
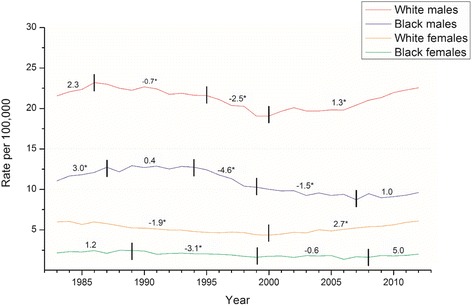
Trends in the age-standardized mortality rate (U.S. 2000 standard population) of suicide in the four populations in the U.S. Vertical lines indicate year in which a joinpoint analysis identifies a change in suicide trends. Annual percentage change in suicide rates are marked in each segment, those with asterisk (*) means significantly different from zero at α = 0.05

Trends of age-standardized mortality rate (ASMR) of suicide by gender and race for the study period of 1983–2012 were shown in Fig. 1. During this period, the white males had the highest ASMR, followed by black males, white females, and black females. The male suicide rates were predominant in both races, with the male to female ratio in ASMR ranged from 3.6 to 4.5 in the white people and from 4.9 to 6.1 in the black people across calendar years. The PACs for specific period segments and APACs for full period in these four population groups were presented in Table 1. Our results showed a significant decrease suicide rate in the four populations in 1990s. We also found a significant increase after 2000 in the white males and females, and a reversed trend in black males and females from negative to positive although they were not significant. We found that all APACs were small and not significant.

**Table 1:** Joinpoint analysis on time trends of age-standardized mortality rates for suicide

Population	Calendar period	PAC(95 % CI)
White males	1983–1986	2.3(−0.3,4.9)
	1986–1995	−0.7(−1.3,–0.2)^a^
	1995–2000	−2.5(−4.1,–0.9)^a^
	2000–2012	1.3(1.0,1.7)^a^
	*Full period*	*APAC = 0.1(−0.3,0.5)*
White females	1983–2000	−1.9(−2.1,-1.7)^a^
	2000–2012	2.7(2.4,3.1)^a^
	*Full period*	*APAC = 0.0(−0.2,0.2)*
Black males	1983–1987	3.0(0.9,5.2)^a^
	1987–1994	0.4(−0.8,1.5)
	1994–1999	−4.6(−6.6,-2.6)^a^
	1999–2007	−1.5(−2.4,-0.7)^a^
	2007–2012	1.0(−0.5,2.5)
	*Full period*	*APAC = −0.6(−1.2,0.0)*
Black females	1983–1989	1.2(−2.4,4.8)
	1989–1999	−3.1(−4.9,-1.2)^a^
	1999–2008	−0.6(−2.9,1.7)
	2008-2012	5.0(−1.7,12.2)
	*Full period*	*APAC = −0.4(−1.8,1.1)*

^a^Significantly different from zero at α = 0.05; APAC: average percent annual change; PAC: percentage of annual change; CI: confidence interval

**Fig. 2:**
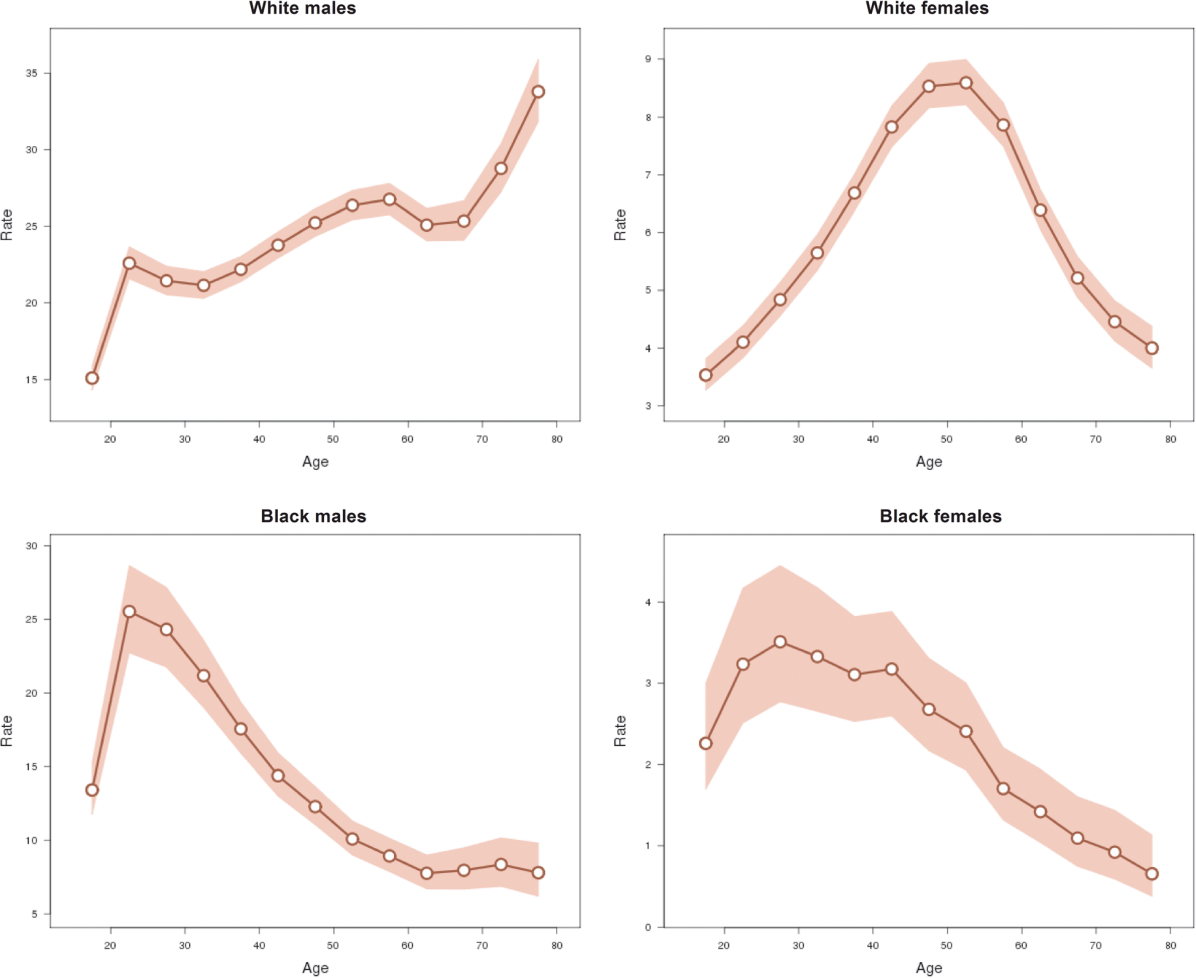
Longitudinal age curves of suicide rate. Longitudinal age curves of the suicide rates (1/100 000) and the corresponding 95 % confidence intervals by gender and race in the U.S

The longitudinal age curves of suicide rate by gender and race in the US were illustrated in Fig. 2. For the white people, the risk of suicide increased generally in males except for minor decreases at ages 20–29 and 55–59 years, while the risk in females increased with age until peaking at ages 45–54 years and showed a decline thereafter. For the black people, the risk of suicide in both genders showed a more similar trend: the risk in males increased with age until peaking at ages 20–24 years and then monotonically declined except for slight increases at old ages (60–74 years), whereas the risk in females increased with age until peaking at ages 25–29 years and then monotonically declined except for slight increases at middle ages (35–44 years).

**Fig. 3:**
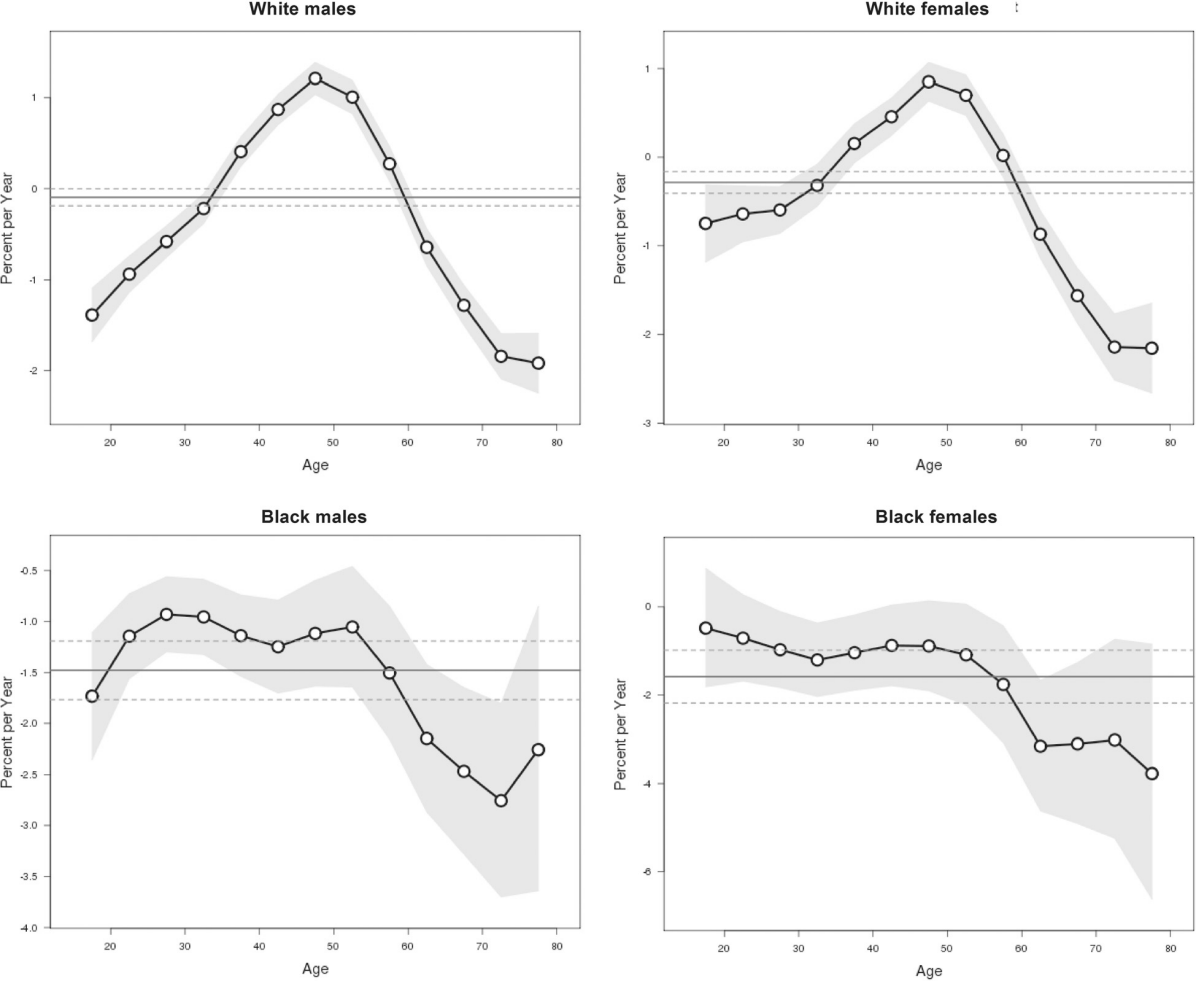
Local drift values of suicide rates. Age group specific annual percent change (%) in the suicide mortality rates and the corresponding 95 % confidence intervals by gender and race in the U.S

The local drift values by gender and race for specific age groups, which indicates the PACs in the suicide rates during the study periods, are displayed in Fig. 3. The local drift values varied greatly across age groups in the white males and females; most of these values were under 0 only with a few significant exceptions at the middle age groups. Specifically, we observed dramatically elevated local drift values at ages 35–59 years in both genders. For the black people, we observed local drift values under 0 in all age groups in both genders, which were higher at ages 20–54 years in males and 15–54 in females.

**Fig. 4:**
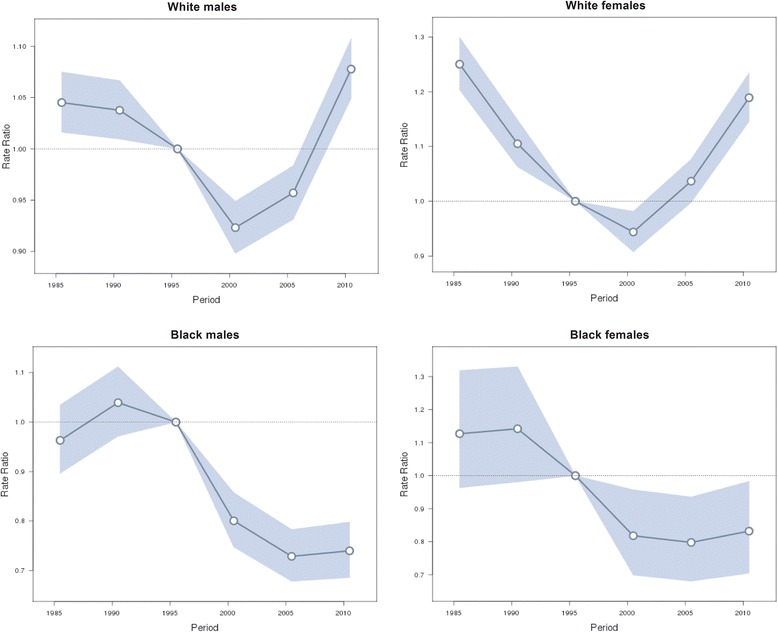
Period effects on suicide rate. Period effects obtained from age-period-cohort analyses for the suicide rates and the corresponding 95 % confidence intervals by gender and race in the U.S

**Fig. 5:**
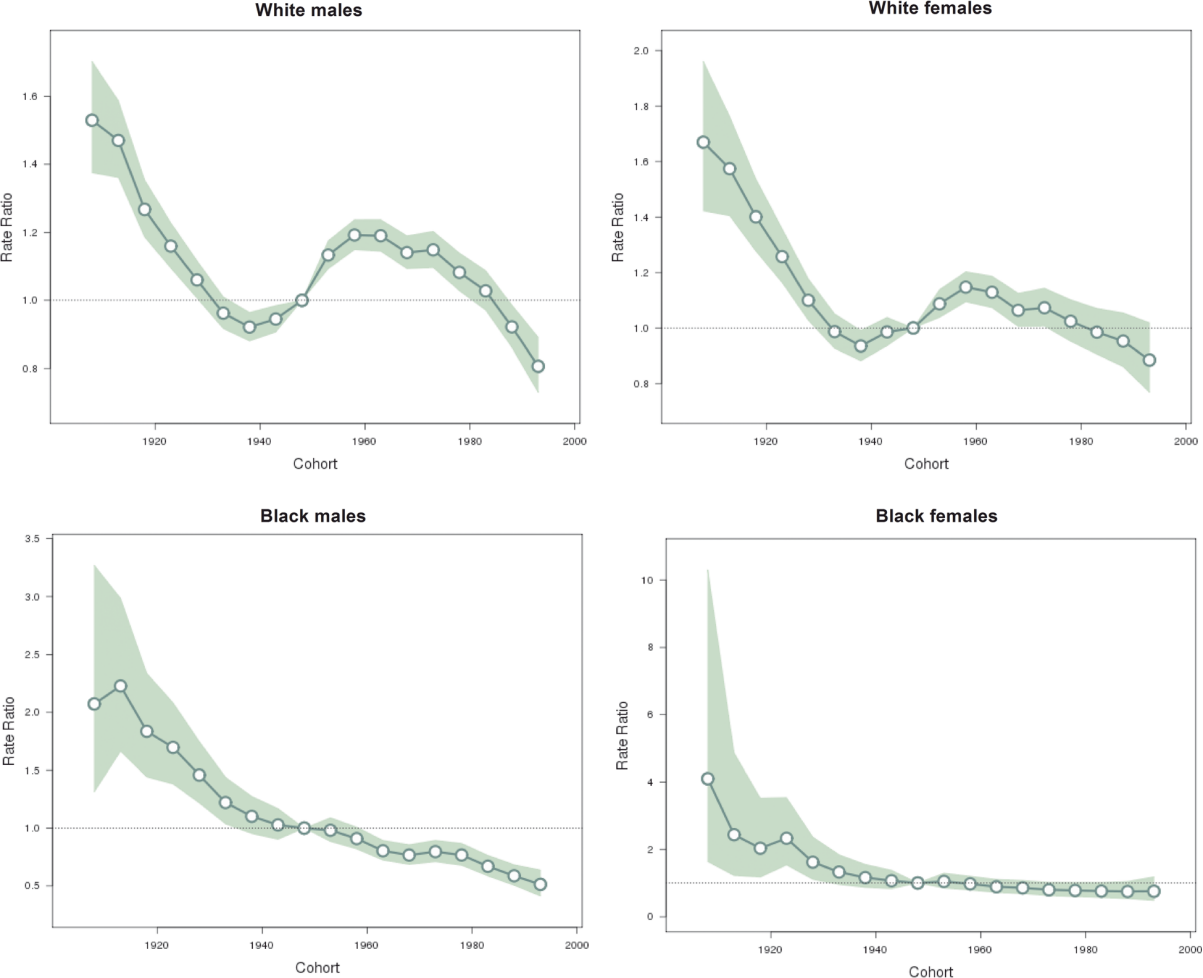
Cohort effects on suicide rate. Cohort effects obtained from age-period-cohort analyses of suicide rates and the corresponding 95 % confidence intervals by gender and race in the U.S

The estimated period and cohort effects by gender and race are displayed in Figs. 4 and 5 respectively. We observed period effects in similar patterns for both sexes for the white people, which showed downward trends since the period of 1983–1987 and then turned upward since 2000 with a significantly elevated risk for the periods after 2005. For the black people, the period effects also showed downward trends in both genders in 1990s, but there was a slight increase before it. In addition, those downward trends slowed after 2000 and shifted to slight upwards since 2005. Cohort effects were found to have similar patterns for both genders for the white people, which decreased monotonically except for the cohorts from 1940–44 to 1960–64. For the black people, the cohort effects in both genders showed a general downward trend.

Using the specific results of Wald tests (not shown), we found statistically significant cohort and period effects for both genders in both white and black populations (*P* < 0.05 for all), and so were the local drifts (*P* < 0.05 for all).

## Discussion

Our study compared trends of suicide mortality between different genders and races in the United States in recent three decades using age, period, and cohort analysis. Although ASMR of suicide in white males, white females, black males, and black females all changed at different degrees from 1983–2012, we found the overall situation almost has not changed from the APACs due to that these changes offset each other to a large extent. Of note, the results of local drifts revealed that the suicide mortality of middle aged white males and females experienced an increasing trend in general, with a peak about 1 % per year near ages 45–49 in both groups.

Suicide risk varies from different age groups due to physiological changes, life experience, social part or status changes, or a blend of these [23]. Identifying high risk groups could contribute to the suicide control and prevention. Back in 1897, Emile Durkheim described a monotonically increasing relationship between age and suicide in his book *Suicide* [25]. Such relationship has been noticed more than once since the start of the 19th century, making it one of the most recognized facts about suicide [26]. However, this monotonic relationship has disappeared and replaced by a more complicated one.

Our findings about the age effect on suicide demonstrate an obvious difference between white males and females in the U.S. Although similar difference in age groups has been reported in previous suicide studies that aimed at all the American males and females, results are limited due to their unadjusted cohort and/or period effects. After controlling for these effects in the APC analysis, we confirmed that the distinction in age-specific suicide rate patterns does exist, with the peak at 75 to 79 for white males and 45 to 54 for white females. Our findings are consistent with Phillips’s APC analysis [12] of the whole American population without separating race, which showed that age curves of suicide for males and females displayed different patterns. This consistence may be related to the fact that the majority of the whole population is the white people (makes up about 70–80 % [27] of the American population during our study period). The reason why suicide risk peaked in old age in white males is probably because retirement, death of relatives (especially spouse) and/or friends, physical limitations to mobility, and serious illness all contribute to more severe isolation in later life [12, 28]. Empirical studies have confirmed a connection between social isolation and inclination toward suicide among old people [28]. However, it seems that physical and/or mood changes due to menopause and “empty-nest crisis” may play more important roles for white women [12], which lead to the suicide peak in the middle age.

With respect to the black people, our results indicated that young people are predominantly susceptible to suicide: risk peaks in early 20s for black males and late 20s for black females. These patterns are very different from the age patterns of the white population and the whole population. These findings suggest that minorities’ age patterns of suicide may have been masked up by the white people in the whole population analysis. The fact that suicide among the black population is a youthful phenomenon is not a new finding [29, 30]. Although the specific reason remains unclear, according to existing studies [26, 30–32], we could speculate that many factors– including lower education and high school dropout; lower socioeconomic status; higher parental divorce rate/single-parent family; early onset of puberty; more likely to exposure to violence and traumatic stress; and less likely to seek help or report for depressive symptoms, suicidal ideation, and suicide attempts – may contribute to this phenomenon.

The cohort effects of the young people and the extremely old must be interpreted with caution due to the few number of observations in both groups and their bigger standard errors than other groups [33]. Accordingly, we concentrated on the general patterns of cohort effect in the middle age range. Our study found that risk by birth cohorts all showed a descending trend except that from cohort 1940 in white males and females, which showed an obvious increase which peaked in around cohort 1960. The declines of cohort effect were also reported in other countries [34] such as European countries and South Korea. This finding is some surprising because these declines suggested a decreasing relative risk of suicide in more recent generations, while trends of suspected risk factors would support an increase in suicide risk in successive cohorts.

General risk factors for suicide identified in previous researches include [35] mental disorders (especially depression and schizophrenia), coincident behavior (shifts between more or less lethal methods; abuse of alcohol and illicit drugs) and sociocultural context (various social cohesions; psychological factors). Although these factors may contribute to the increased cohort effect in the white people, they were less likely to be the driver of the rest decreases of birth effects. While there is still some question about the reasons for these declines, improvements in health care—both in treatment and accessibility for mental and substance abuse disorders, improvement of the level of education for all, and increasing awareness of suicide among the public are likely explanations for them, at least for the recent cohort declines. Increased education could also improve abilities in problem tackling, conflict resolution, and skills for managing disputes, which are protective factors. More knowledge on suicide could improve help-seeking from the family, friends or specialists.

Since different populations, different study periods, or even different statistical methods [36] may result in different cohort effects, it is often hard to indirectly compare cohort results among similar studies. But even so, many studies in Western countries have observed increased suicide risks in successive cohorts in the post-war period [37, 38]. Two studies [12, 39] in the U.S. population found this increasing effect in those who were born after around 1940. However, contrary to our results, these two studies reported that this increase trend continued to the cohort 1990 and they showed a weaker period effect after 2000 or did not find any period effect on trends in the U.S. suicide rates.

Although the age and cohort effects are relatively strong, the period effect appeared to be a more primary factor in the U.S. suicide trend according to the previous Joinpoint results. From Fig. 4, we can see that there was a similar period effect trend between different genders in the same race group. But between different race groups, differences were found in the period before 1990 and after 2000.

One thing that could influence suicide rates in certain calendar years across all age groups is the economy conditions [40–42]. By and large, the U.S. economy flourished amid the 1990s, with correspondingly lower unemployment rates [43], coincided with the time all gender and race groups experienced a downward trend of period effect. Based on this reasoning, if economic thriving of the 1990s was a major factor affecting suicide, the late economic downturn could be expected to cause increasing suicide death rates. We found that from 2000 to 2005, suicide risk for the white people stopped decreasing and began to increase, whereas for the black people, the decreased trend began to slow down. From 2005–2010, suicide risk in the white people increased more dramatically and the black people’s risk reversed trend to incline marginally. It seems that period effects changed in line with the economy condition since it suffered a recession in early 2000s and then experienced the Financial Crisis of 2007–08. So we have reason to suggest that economy conditions have association with the period effect of suicide.

We found that the slight increase in period effects of the black people before 1990 is intriguing. We speculate that it was likely to be caused by the deindustrialization in 1980s, whose impact disproportionately burdened Black families and communities [44]. We also found that the period effect from 2001–2010 was more apparent in the white people than in the black. This race difference might be explained by the difference in race susceptibility to socioeconomic factors, for instance, economic depression.

Socioeconomic factors could also influence certain cohort effects because those in middle age may be more vulnerable to economic stress [41, 45]. But in our cohort effect results mentioned above, the obvious increase with peaks in cohorts around 1960 (middle aged people during Financial Crisis of 2007–08) was only found in white males and females. It seems that the crisis has almost no more influence on the black middle aged people at that time compared to their white counterparts.

Like other APC analysis studies, the major limitation of the present study was the inevitability of being affected by ecological fallacy since interpretations from results at population levels do not necessarily hold for individuals. Therefore, all hypotheses raised in this study still need further confirmation in the future individual-based studies. In addition, improvements in the accuracy and completeness of suicide rate data may lead to bias for temporal analysis. However, it was unlikely that major patterns based on these statistics were in error [46], for example, the downward trend in 1990s and the upward trend in 2000s.

## Conclusions

In summary, our study showed that, although ASMR of suicide in all four populations changed at different degrees from 1983 to 2012, the overall situation almost has not changed since these changes offset each other. The distinction in age-specific suicide rate patterns does exist by gender and by race after controlling for period and cohort effects, which suggests that minorities’ age patterns of suicide may have been masked up by the white people in the whole population. The differences of period effects and cohort effects between white and black Americans were likely to be mainly explained by the difference in race susceptibility to economic depression.

## Abbreviations

APAC, average percent annual change; APC analysis, age-period-cohort analysis; ASMR, age-standardized mortality rate; CDC, Centers for Disease Control and Prevention; PAC, percentage of annual change; WISQARS, Web-based Injury Statistics Query and Reporting System.

## References

[1] World Health Organization. Public health action for the prevention of suicide: a framework. Geneva: World Health Organization; 2012.

[2] Hu G, Wilcox HC, Wissow L, Baker SP (2008). Mid-life suicide: an increasing problem in US Whites, 1999–2005. Am J Prev Med.

[3] Curtin SC, Warner M, Hedegaard H. Increase in suicide in the United States, 1999–2014. NCHS Data Brief. Hyattsville, MD: National Center for Health Statistics. 2016;241:1–8. 27111185

[4] Control CfD, Prevention. Web-based Injury Statistics Query and Reporting System (WISQARS)(2010). National Center for Injury Prevention and Control, CDC (producer) Available at: www.cdc.gov/injury/wisqars/index.html. Accessed March 2011, 13.

[5] Curtin SC, Warner M, Hedegaard M (2016). Suicide Rates for Females and Males by Race and Ethnicity: United States, 1999 and 2014.

[6] Rockett IR, Regier MD, Kapusta ND, Coben JH, Miller TR, Hanzlick RL, Todd KH, Sattin RW, Kennedy LW, Kleinig J (2012). Leading causes of unintentional and intentional injury mortality: United States, 2000–2009. Am J Public Health.

[7] Surtees PG, Duffy J (1989). Suicide in England and Wales 1946–1985: an age-period-cohort analysis. Acta Psychiatr Scand.

[8] Wang Z, Bao J, Yu C, Wang J, Li C (2015). Secular Trends of Breast Cancer in China, South Korea, Japan and the United States: Application of the Age-Period-Cohort Analysis. Int J Environ Res Public Health.

[9] Odagiri Y, Uchida H, Nakano M (2009). Gender differences in age, period, and birth-cohort effect on suicide mortality rate in Japan 1985–2006. Asia Pac J Public Health.

[10] Granizo JJ, Guallar E, Rodriguez-Artalejo F (1996). Age-period-cohort analysis of suicide mortality rates in Spain, 1959–1991. Int J Epidemiol.

[11] Allebeck P, Brandt L, Nordstrom P, Åsgård U (1996). Are suicide trends among the young reversing? Age, period and cohort analyses of suicide rates in Sweden. Acta Psychiatr Scand.

[12] Phillips JA (2014). A changing epidemiology of suicide? The influence of birth cohorts on suicide rates in the United States. Soc Sci Med.

[13] Nock MK, Borges G, Bromet EJ, Cha CB, Kessler RC, Lee S (2008). Suicide and suicidal behavior. Epidemiol Rev.

[14] McIntosh JL (1989). Trends in racial differences in US suicide statistics. Death Stud.

[15] Woodbury MA, Manton KG, Blazer D (1988). Trends in US suicide mortality rates 1968 to 1982: Race and sex differences in age, period and cohort components. Int J Epidemiol.

[16] Control CfD. Injury prevention & control: data & statistics (WISQARS). Welcome to WISQARS (Web-based Injury Statistics Query and Reporting System). CDC Web site: http://www.cdc.gov/injury/wisqars/. Accessed May 15 2014.

[17] Control CfD (1989). Mortality data from the National Vital Statistics System. MMWR Morb Mortal Wkly Rep.

[18] Anderson RN, Miniño AM, Hoyert DL, Rosenberg HM (2001). Comparability of cause of death between ICD-9 and ICD-10: preliminary estimates. Nat Vital Stat Rep.

[19] Liaw Y-P, Huang Y-C, Lien G-W (2005). Patterns of lung cancer mortality in 23 countries: application of the age-period-cohort model. BMC Public Health.

[20] Su S-Y, Huang J-Y, Jian Z-H, Ho C-C, Lung C-C, Liaw Y-P (2012). Mortality of colorectal cancer in Taiwan, 1971–2010: temporal changes and age–period–cohort analysis. Int J Color Dis.

[21] Anderson RN, Rosenberg HM (1998). Age standardization of death rates: implementation of the year 2000 standard. Natl Vital Stat Rep.

[22] Clegg LX, Hankey BF, Tiwari R, Feuer EJ, Edwards BK (2009). Estimating average annual per cent change in trend analysis. Stat Med.

[23] Yang Y, Land KC. Age-period-cohort analysis: New models, methods, and empirical applications. Boca Raton: CRC Press; 2013.

[24] Rosenberg PS, Check DP, Anderson WF (2014). A Web Tool for Age–Period–Cohort Analysis of Cancer Incidence and Mortality Rates. Cancer Epidemiol Biomark Prev.

[25] Durkheim E (1951). Suicide: A study in sociology. Translated by JA Spaulding and G. Simpson.

[26] Cutler DM, Glaeser EL, Norberg KE. Explaining the rise in youth suicide. In: Risky behavior among youths: An economic analysis. Chicago: University of Chicago Press; 2001:219–270.

[27] Humes K, Jones NA, Ramirez RR. Overview of race and Hispanic origin, 2010. Washington: US Department of Commerce, Economics and Statistics Administration, US Census Bureau; 2011.

[28] Stack S. Social correlates of suicide by age. In: Life span perspectives of suicide. Berlin: Springer; 1991:187–213.

[29] Gibbs JT (1988). Conceptual, methodological, and sociocultural issues in black youth suicide: Implications for assessment and early intervention. Suicide Life Threat Behav.

[30] Crosby AE, Molock SD (2006). Introduction: Suicidal behaviors in the African American community. J Black Psychol.

[31] Balis T, Postolache TT (2008). Ethnic differences in adolescent suicide in the United States. Int J Child Health Human Dev.

[32] Aseltine RH, DeMartino R (2004). An outcome evaluation of the SOS suicide prevention program. Am J Public Health.

[33] Yang Y, Schulhofer-Wohl S, Fu WJ, Land KC (2008). The Intrinsic Estimator for Age-Period-Cohort Analysis: What It Is and How to Use It1. Am J Sociol.

[34] Park C, Jee YH, Jung KJ (2016). Age-Period-Cohort Analysis of the Suicide Rate in Korea. J Affect Disord..

[35] Bopp M, Gostynski M, Lauber C, Gutzwiller F, Rössler W (2006). Age–period–cohort analysis of Swiss suicide data, 1881–2000. Eur Arch Psychiatry Clin Neurosci.

[36] Keyes KM, Utz RL, Robinson W, Li G (2010). What is a cohort effect? Comparison of three statistical methods for modeling cohort effects in obesity prevalence in the United States, 1971–2006. Soc Sci Med.

[37] Gunnell D, Middleton N, Whitley E, Dorling D, Frankel S (2003). Influence of cohort effects on patterns of suicide in England and Wales, 1950–1999. Br J Psychiatry.

[38] Chung RY, Yip BH, Chan SS, Wong S. Cohort effects of suicide mortality are sex specific in the rapidly developed Hong Kong Chinese population, 1976–2010. Depression and anxiety. 2015;33:558–566. 10.1002/da.22431 26414148

[39] Keyes KM, Li G. Age–Period–Cohort Modeling. In: Injury Research. Springer; 2012: 409–426.

[40] Hawton K, Haw C (2013). Economic recession and suicide.

[41] Reeves A, Stuckler D, McKee M, Gunnell D, Chang S-S, Basu S (2012). Increase in state suicide rates in the USA during economic recession. Lancet.

[42] Eyer J (1976). Prosperity as a cause of death. Int J Health Serv.

[43] McKeown RE, Cuffe SP, Schulz RM (2006). US suicide rates by age group, 1970–2002: an examination of recent trends. Am J Public Health.

[44] Joe S (2006). Explaining changes in the patterns of Black suicide in the United States from 1981 to 2002: An age, cohort, and period analysis. J Black Psychol.

[45] Phillips JA, Robin AV, Nugent CN, Idler EL. Understanding recent changes in suicide rates among the middle-aged: period or cohort effects? Public Health Rep. 2010;125:680–8. 10.1177/003335491012500510 PMC2925004 20873284

[46] O’Carroll PW (1989). A consideration of the validity and reliability of suicide mortality data. Suicide Life Threat Behav.

